# Unexpected Infection with *Armillifer* Parasites

**DOI:** 10.3201/eid2312.171189

**Published:** 2017-12

**Authors:** Idzi Potters, Claude Desaive, Steven Van Den Broucke, Marjan Van Esbroeck, Lutgarde Lynen

**Affiliations:** Institute of Tropical Medicine Antwerp, Antwerp, Belgium (I. Potters, S. Van Den Broucke, M. Van Esbroeck, L. Lynen);; Central University Hospital of Liège, Liège, Belgium (C. Desaive)

**Keywords:** Armillifer, pentastomida, snakes, parasites, surgery, Africa

## Abstract

Visceral pentastomiasis is usually found incidentally during surgery. We describe a case of visceral pentastomiasis discovered during inguinoscrotal hernia surgery for a man from Benin, Africa. Because surgical removal of nymphs is needed for symptomatic patients only, this patient’s asymptomatic pentastomiasis was not treated and he recovered from surgery uneventfully.

In November 2015, a surgeon from Belgium, working for Medics without Vacation in Bassila, Benin, Africa, incidentally discovered pentastomiasis in an adult man during surgery for a massive inguinoscrotal hernia (half a liter content). Other than the hernia, the patient had no health problems. During the procedure, the surgeon observed at least 10 coiled, larva-like structures on the patient’s peritoneal tissue. He removed the hernial sac and sent a tissue specimen to the national reference laboratory for parasitology at the Institute of Tropical Medicine (Antwerp, Belgium) for identification of the parasite. Apart from the hernia symptoms, the patient was asymptomatic, so the parasites were not removed; the patient’s surgical recovery was uneventful.

Macroscopic examination of the peritoneal tissue detected 8 distinct, typical larva-like structures with an average length of 1–2 cm (Figure, panel A). Because the structures were suspected to be pentastomes, they were compared with reference material from the Institute of Tropical Medicine Educational Department (Figure, panel B) and confirmed as *Armillifer* spp. nymphs. On the basis of the patient’s residence in Benin, and the fact that the recovered nymphs consistently exhibited <22 annuli, the structures were presumptively identified as *A. armillatus* (*1**,**2*). Adults of this species are often found in the respiratory system of large snakes. Although no information was available regarding the patient’s contact with snakes, the presence of this parasite in a resident of Benin is not surprising because snake consumption in that country is common practice. The patient’s surgeon confirmed that dead snakes were indeed often sold for consumption along the streets near the hospital.

**Figure:**
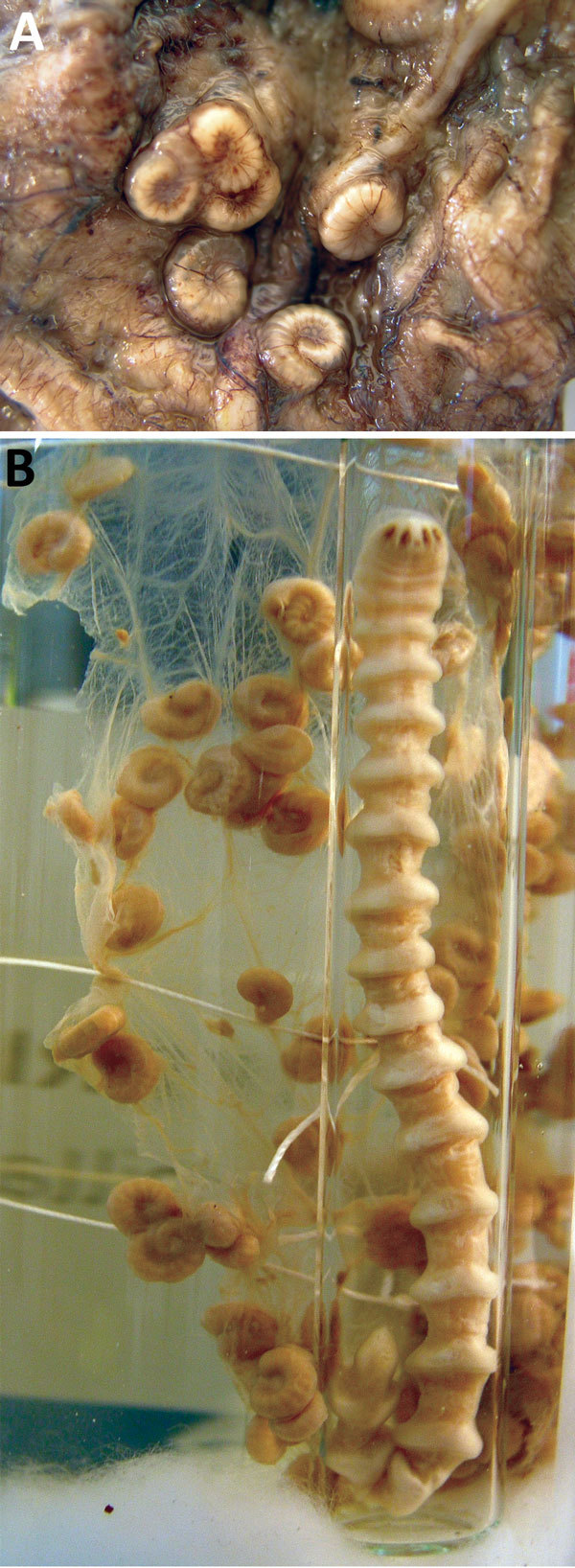
*Armillifer armillatus* parasites. A) Typically coiled *Armillifer armillatus* nymphs, averaging 1–2 cm long and consistently showing <22 annuli. B) Adult female and numerous nymphs. Reference material from the Educational Department, Institute of Tropical Medicine, Antwerp, Belgium.

The Pentastomida are a peculiar group of gonochoric, vermiform endoparasites, currently classified as a unique phylum, related to branchiuran crustaceans (*3*). The main characteristics of this group of ancient parasitic arthropods are an often annulated elongate body and a mouth typically flanked by 2 pairs of hooks. 

Human visceral pentastomiasis can be caused by several species of pentastomes: *Linguatula serrata* (worldwide, predominantly the Middle East), *A. armillatus* (West and Central Africa), *A. moniliformis* (Southeast Asia), *A. grandis* (Africa), *A. agkistrodontis* (China), *Porocephalus crotali* (worldwide, predominantly the Americas), and *P. taiwana* (China) (*2**,**4**,**5*). Reported cases of human visceral pentastomiasis were caused mainly by *A. armillatus* pentastomes (*6*) from infected snakes, which shed ova in excretions and respiratory secretions, thereby contaminating vegetation and water. Intermediate hosts are mainly rodents and small mammals. After oral uptake by the intermediate host, the ova hatch and free 4-legged primary larvae that migrate to the viscera, become encapsulated, and after several molts transform into legless nymphs (*2*,*7*). The cycle is complete when the intermediate host is in turn consumed by a snake of an appropriate species. Humans can become intermediate hosts through ingestion of environmental ova, by eating undercooked infected snake meat, or by oral uptake of ova when in close contact with snakes (*1**,**2**,**6*). Diagnosis of human visceral pentastomiasis is classically made by either identifying the nymphs during autopsy or surgery or by discovering typically crescentic or coiled opacities, representing calcified dead larvae, on abdominal or chest radiographs. 

The finding of pentastomiasis in patients with inguinal hernia, as with the patient reported here, has been described for 2 patients in Ghana (*8*). The number of reported cases of human pentastomiasis from Africa is increasing (*6**,**8*); because infection with *Armillifer* parasites is usually asymptomatic, we assume that the incidence of this infection is underestimated. Autopsy studies have reported prevalence rates as high as 22% in the Democratic Republic of the Congo and 45% in Malaysia (*9**,**10*). As industrialized countries are increasingly receiving immigrants and refugees from Africa, it is conceivable that this disease will be more frequently encountered.

Because pentastomes are usually not mentioned in the helminth section of parasitology textbooks, laboratory technicians, clinicians, and surgeons who are unfamiliar with this parasite might be startled when incidentally discovering it. They should note that visceral pentastomiasis is usually asymptomatic and that surgical removal of the nymphs should be considered only for symptomatic patients with high parasite loads. No medical treatment is available.
